# It Is All About Location: Smartphones and Tracking the Spread of COVID-19

**DOI:** 10.1177/2056305120948257

**Published:** 2020-07-30

**Authors:** Jordan Frith, Michael Saker

**Affiliations:** 1 Clemson University, USA; 2 City, University of London, UK

**Keywords:** smartphones, locative media, surveillance, privacy, COVID-19

## Abstract

Mobile phone location data have become tied to understandings of and responses to the COVID-19 pandemic. Data visualizations have used mobile phone data to inform people about how mobility practices may be linked to the spread of the virus, and governments have explored contact tracing that relies upon mobile phone data. This article examines how these uses of location data implicate three particular issues that have been present in the growing body of locative media research: (1) anonymized data are often not anonymous, (2) location data are not always representative and can exacerbate inequality, and (3) location data are a key part of the extension of the surveillance state.

It is a strange feeling when something you have been researching for almost a decade suddenly becomes a part of the public conversation. That happened to the two of us when discussions of COVID-19 started intersecting with mobile phone location data. We have studied mobile phone location data since we were doctoral students, and we have argued that institutions should be more transparent about how much location data they collect (Evans & Saker, 2017; Frith, 2015). Somewhat surprisingly, it took a global pandemic to drive home that point.

Within a few weeks of the virus’ spread in the United States, discussions of location data were everywhere; visualizations of mobility practices became a recurring feature of media outlets, and governments began discussing the use of location data to track the mobility of infected people. This essay argues location data’s 15 minutes of public fame is a potential opportunity to raise attention about locational privacy issues that were present long before COVID-19. Namely, we look at three major locational data issues implicated by the current pandemic: (1) anonymized data sometimes are not anonymous, (2) locational data are often not representative and can exacerbate inequality, and (3) location data are a key part of the extension of the surveillance state. We conclude by asking whether this moment of visibility for location data collection could provide an opportunity to push for new media literacies.

Mobile phones have transmitted location data since the advent of cell towers (Ling & Donner, 2008). However, the accuracy and the number of actors who collect location data have shifted. Actors—ranging from telecoms to Google and Apple to individual app developers and whoever they sell data to—collect location data produced through mobile phones, and people have little recourse to understand how their data are being used and who has access to them (Frith, 2015). Take as an example the recent visualizations of cell phone location data used to show everything from the travel patterns of spring breakers to the distance people traveled in mid-March after stay-at-home orders were given; however, people were left guessing where the data came from. Regarding the spring break visualization, for example, CNN was unable to track down the origin of these data (Barrett, 2020). A series of *New York Times* visualizations that tracked movement in relation to stay-at-home orders reported they received their data from an intelligence firm called Cuebiq (Glanz et al., 2020), which raises questions about where Cuebiq got these data, and so on.

Data visualizations are just one example of the increase in attention given to location data during the pandemic. A more prominent example is government (and sometimes corporate) digital contact tracing. Digital contact tracing involves identifying infected individuals and then tracing the people they have been in contact with. For the most part, this process uses Bluetooth technology that logs when devices are in close proximity to another device associated with an infected user for a prolonged period of time. Contact tracing applications feature different design choices and sources of data, and these choices—for example, whether the data storage is decentralized or whether the Bluetooth data are combined with GPS—raise different levels of privacy concerns (Bell, 2020). Specific systems aside, digital contact tracing raises important questions about issues such as locational privacy and digital inequality that should be addressed, especially because some experts think these apps will neither be particularly effective nor sufficiently adopted to render them useful (Alkhatib, 2020; Bell, 2020).

The locative functions of smartphones have been a major focus of the field of Mobile Communication Studies (MCS) for more than a decade (Campbell, 2018), and much of that research has focused on privacy issues (de Souza e Silva & Frith, 2010). While COVID-19 does not raise wholly novel locational privacy issues, it does increase the public visibility of issues associated with locational data. By no means is the list below comprehensive, but we tackle three of those issues and conclude by speculating about whether the pandemic could be an opportunity to raise awareness of the extent and consequences of the location data our smartphones transmit.

## Issue 1: Anonymized Data Are Often Not Anonymous

The COVID-19 pandemic has led to an explosion of popular science communication, such as the data visualizations detailed above. These visualizations use anonymized mobile phone data to visualize people’s mobility. The spring break visualization, for example, put anonymized dots on a map and visualized how they spread across the United States as they left Florida. The data are scrubbed of individual identifiers, which supposedly make it acceptable from a privacy standpoint. However, these visualizations and the broader use of anonymized data raise serious issues about how effective anonymization is.

In the mid-2000s, America Online released a huge amount of anonymized search data to the public. Through only a handful of search queries, researchers were able to deanonymize some of the data and track it back to individual users (Barbaro & Zeller, 2006). And the same has been shown with location data: one study took an anonymized location dataset and found that “four spatio-temporal points are enough to uniquely identify 95% of the individuals” (de Montjoye et al., 2013, p. 1). Consequently, the anonymized data visualizations used to visualize mobility in relation to the pandemic could raise similar issues and work as a conversation starter about how identifiable apparently “anonymous” location data actually are.

## Issue 2: Location Data Are Not Always Representative and Can Exacerbate Inequality

One of the problems with the occasionally blind faith in big data has been ignorance of just what data are collected and who it leaves out (boyd & Crawford, 2012). That has always been true for location data as well. Back in the early 2010s, the *LiveHoods* project produced maps of Foursquare check-ins, and some outlets used these maps to show the “popular” areas of the cities. However, the maps were markedly devoid of check-ins in large parts of the city. Were those areas empty? No. Instead, they were often minority areas of the city where people did not use Foursquare (Frith, 2017). As locative media research has shown, context and marginalization matters with location data (Graham & Zook, 2013).

Digital contact tracing will likely exacerbate the inequality already present in locational data analyses. Most of these apps work by using Bluetooth to record the proximity between handsets so people can be alerted if someone gets sick. Obviously, people can only be alerted if they own a smartphone. Consequently, children, the homeless, some elderly, non-adopters, and people without financial means will be left out of the analysis (Alkhatib, 2020). Even many people who do own smartphones will not be able to participate because their devices do not have the proper low-energy Bluetooth capabilities. Even if these people wanted to participate in digital contact tracing, they will not be able to and will instead be rendered invisible in the analysis, with consequences possibly far more serious than the neighborhoods left out of Foursquare maps.

## Issue 3: Location Data Are a Key Part of the Extension of the Surveillance State

Some of the very early writing about locative media—mostly from artists—discussed its surveillance potential (Tuters & Varnellis, 2006). Knowing where a device is or what it is close to raises obvious surveillance concerns. And much of the locative media literature has tracked how surveillance has become more extensive as smartphones have become more widely adopted. After all, locational metadata played a role in Edward Snowden’s revelations, and the ability to track location is a major piece of the larger surveillance state (Frith, 2015).

As many activists have warned, the embrace of digital contact tracing could involve strengthening the surveillance state in unprecedented ways, which is why privacy protections are so important (Bell, 2020). And the history of surveillance suggests it can be difficult to roll back surveillance technologies once they have been enacted (Lyon, 2010). Much of the surveillance state built after 9/11 remains in effect almost two decades after the attack. Maybe the location data collection authorized through contact tracing would be removed once the pandemic passed. Just as likely, however, is that these systems remain in place to deal with the “next” outbreak, a possibility some privacy advocates fear (Alkhatib, 2020). And the longer these systems remain in place, the more entrenched they inevitably become, and the more uses they will likely accrue.

## The Pandemic and Media Literacies

Looking for any silver linings amid a global pandemic seems almost crass, but the rise of popular data visualizations and digital contact tracing could be an opportunity to increase people’s knowledge about the extent of data smartphones share about their lives. We recognize that improved media literacies around these issues are only one small part of a larger puzzle. As media scholars have argued, many issues that can supposedly be solved by improved media literacies need to be addressed at the level of policy to be effective (Vaidhyanathan, 2018). That is certainly true of digital contact tracing as well. The policy and architectural levels are where decisions about what to collect, where to store the data, and how the data can be used will be made. However, an expanded knowledge of how smartphones work and questions about where data come from can help people make decisions when choosing how to engage with these apps and their smartphones more generally. They can also become pedagogical tools within media classrooms. Students can track down where popular mobile phone visualizations got their data (and often end up at dead ends). They can learn about the deanonymization of datasets and the broader access and marginalization issues present in projects like digital contact tracing.

While these literacies will not solve the questions surrounding digital contact tracing, they can help people engage differently with personal decisions about whether to use these apps and to ask questions about how they work. Smartphones constantly transmit location data, which can then make its way into various datasets without their knowledge. Those datasets might be anonymized, but that is often not as foolproof as the word makes it sound. Contact tracing apps create logs of who people are near, which has major surveillance potential that might never disappear once the apps are created. And on top of those concerns, locative datasets leave out certain users and can essentially render people invisible if they are not using the right technologies.

At this point, it is debatable how successful contact tracing will even be in combating the virus. But what is not debatable is that they, along with various COVID-19 visualizations, raise important questions that have long been present in the locative media literature. We hope that by raising these issues now, the increased attention paid to locative data could be an opportunity to improve media literacies in the future. In particular, these visualizations and contact tracing apps could be valuable pedagogical tools to get students and members of the general public to think more deeply about just how revealing, or not, a picture their smartphones paint of their everyday lives.

## References

[bibr1-2056305120948257] Alkhatib A. (2020, 5 1). We need to talk about digital contact tracing. Ali-Alkhatib.com. https://ali-alkhatib.com/blog/digital-contact-tracing

[bibr2-2056305120948257] Barbaro M. Zeller T. (2006, 8 9). A face is exposed for AOL searcher No. 4417749. The New York Times. https://www.nytimes.com/2006/08/09/technology/09aol.html

[bibr3-2056305120948257] Barrett D. (2020, 4 4). How the cell phones of spring breakers who flouted coronavirus warnings were tracked. CNN. https://www.cnn.com/2020/04/04/tech/location-tracking-florida-coronavirus/index.html

[bibr4-2056305120948257] Bell G. (2020, 4 12). We need mass surveillance to fight COVID-19—But it doesn’t have to be creepy. MIT Technology Review. https://www.technologyreview.com/2020/04/12/999186/covid-19-contact-tracing-surveillance-data-privacy-anonymity/

[bibr5-2056305120948257] boyd d. Crawford K. (2012). Critical questions for Big Data. Information, Communication & Society, 15(5), 662–679. 10.1080/1369118X.2012.678878

[bibr6-2056305120948257] Campbell S. W. (2018). From frontier to field: Old and new theoretical directions in Mobile Communication Studies. Communication Theory, 29(1), 46–65. 10.1093/ct/qty021

[bibr7-2056305120948257] de Montjoye Y.-A. Hidalgo C. A. Verleysen M. Blondel V. D. (2013). Unique in the crowd: The privacy bounds of human mobility. Scientific Reports, 3(1), 1376 10.1038/srep01376 PMC3607247 23524645

[bibr8-2056305120948257] de Souza e Silva A. Frith J. (2010). Locational privacy in public spaces: Media discourses on location-aware mobile technologies. Communication, Culture & Critique, 3(4), 503–525.

[bibr9-2056305120948257] Evans L. Saker M. (2017). Location-based social media: Space, time and identity. Springer.

[bibr10-2056305120948257] Frith J. (2015). Smartphones as locative media. Polity Press.

[bibr11-2056305120948257] Frith J. (2017). Invisibility through the interface: The social consequences of spatial search. Media, Culture & Society, 39(4), 536–551. 10.1177/0163443717698871

[bibr12-2056305120948257] Glanz J. Carey B. Holder J. Watkins D. Valentino-DeVries J. Rojas R. Leatherby L. (2020, 4 2). Where America didn’t stay home even as the virus spread. The New York Times. https://www.nytimes.com/interactive/2020/04/02/us/coronavirus-social-distancing.html

[bibr13-2056305120948257] Graham M. Zook M. (2013). Augmented realities and uneven geographies: Exploring the geolinguistic contours of the web. Environment and Planning A, 45, 77–99.

[bibr14-2056305120948257] Ling R. Donner J. (2008). Mobile phones and mobile communication. Polity Press.

[bibr15-2056305120948257] Lyon D. (2010). Liquid surveillance: The contribution of Zygmunt Bauman to surveillance studies. International Political Sociology, 4, 325–338.

[bibr16-2056305120948257] Tuters M. Varnellis K. (2006). Beyond locative media: Giving shape to the Internet of Things. Leonardo, 39(4), 357–363.

[bibr17-2056305120948257] Vaidhyanathan S. (2018). Antisocial media: How Facebook disconnects us and undermines democracy. Oxford University Press.

